# Late Chronic Left Ventricular Pseudoaneurysm Thrombosis

**DOI:** 10.1016/j.jaccas.2025.104425

**Published:** 2025-08-06

**Authors:** Jacopo Lin, Matteo Saccocci, Eren Cetinel, Francesco Ancona, Giulia Geremia, Ottavio Alfieri, Francesco Maisano, Michele De Bonis

**Affiliations:** a School of Medicine, Vita-Salute San Raffaele University, Milan, Italy; b Department of Cardiac Surgery, IRCCS San Raffaele Hospital, Vita-Salute San Raffaele Scientific Institute, Milan, Italy; c Cardiovascular Imaging Unit, IRCCS San Raffaele Hospital, Milan, Italy; d Outpatient Cardiology Service, San Francesco Clinic, Verona, Italy

**Keywords:** cardiovascular disease, chronic heart failure, left ventricle, myocardial infarction, thrombus, thrombosis, treatment

## Abstract

**Background:**

Left ventricular pseudoaneurysm (LVPA) is a rare but serious complication following myocardial infarction, often leading to thrombus formation and hemodynamic collapse.

**Case Summary:**

We present 2 cases of LVPA in patients with a history of myocardial infarction. A 61-year-old man underwent surgical repair for a large apical LVPA with significant thrombus formation, and a 77-year-old woman with a posterolateral LVPA was treated with percutaneous exclusion. Echocardiography and cardiac computed tomography were used in the diagnosis in both cases, highlighting the delayed presentation of LVPA.

**Discussion:**

LVPA, though rare, poses a high risk for rupture and thrombotic complications. Early recognition is crucial for timely intervention. These cases are of interest owing to the large size of the pseudoaneurysms and the distinct therapeutic approaches used.

**Take-Home Message:**

LVPA can manifest years after myocardial infarction, and timely intervention, whether surgical or percutaneous, is key to improving outcomes in such cases.

## Graphical Abstract

**Figure:**
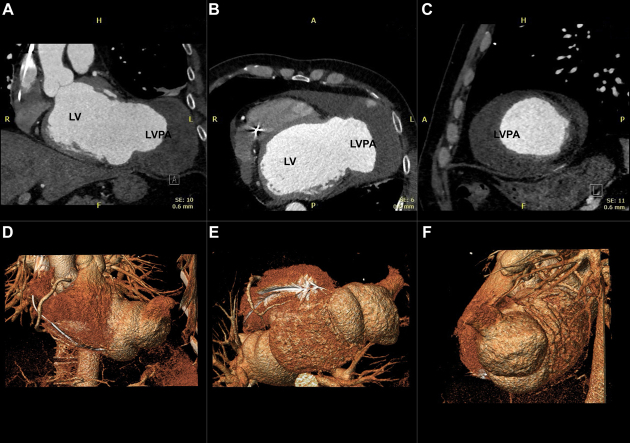

Left ventricular pseudoaneurysm (LVPA) is a rare complication of ventricular free wall rupture following acute myocardial infarction (AMI). Characterized by a narrow pericardial defect, a slender neck is formed that separates the pseudoaneurysmal cavity from the left ventricle (LV).^1^^,^^2^ The neck-to-diameter ratio ranges from 0.25 to 0.50, distinguishing it from true aneurysms, which have a broader connection to the LV.^2^ In contrast to true aneurysms, LVPA lacks myocardial tissue in its wall and is typically contained by pericardial adhesions or epicardial thrombi. Although it may remain asymptomatic, its natural course carries a high risk of rupture, often leading to acute hemodynamic collapse and death.^1^^,^^2^ Other serious complications include thrombus formation within the left ventricular cavity that is due to scarred myocardium and, in rare cases, complete thrombotic obstruction of the LV.^3^ Surgical intervention remains the gold standard, as patients with subacute or contained ruptures have a limited window for emergency treatment, underscoring the need for early recognition to prevent delays in care.^2^ In this report, we discuss 2 remarkable cases of large LVPA that were managed through different therapeutic approaches: one treated surgically and the other via transcatheter intervention.

**Take-Home Messages**

• These cases highlight 2 key points: the delayed management of a potentially fatal complication—both cases were treated 7 years after AMI—and the exceptional size of the LVPA and thrombotic mass, a condition rarely reported in the literature.
• Clinicians should be aware of the potential for delayed presentations of LVPA, particularly in patients with a history of myocardial infarction, and consider early intervention even in atypical or asymptomatic cases to prevent adverse outcomes associated with the unstable nature of the LVPA.

## Patient 1: Case Presentation

A 61-year-old man, NYHA functional class II, presented with nonradiating chest pain associated with mild dyspnea. His medical history was notable for severe single-vessel coronary artery disease with chronic occlusion of the left anterior descending artery, compensated by collateral circulation.

The electrocardiogram showed signs of a previous myocardial infarction in the inferior and anterolateral leads. Owing to the presence of severe left ventricular dysfunction, besides medical therapy, an implantable cardioverter-defibrillator was implanted for primary prevention.

Seven years later, the patient was readmitted for acute heart failure, presenting only with dyspnea at admission. Transthoracic echocardiography (TTE) demonstrated a large left ventricular apical pseudoaneurysm, with evidence of an organized and layered thrombotic mass within it (Video 1). The heart demonstrated severe left ventricular dilation and significantly reduced global systolic function, with an ejection fraction (EF) of 27%. Additionally, there was marked left atrial dilation, grade III diastolic dysfunction, and mild mitral regurgitation.

**Figure 1:**
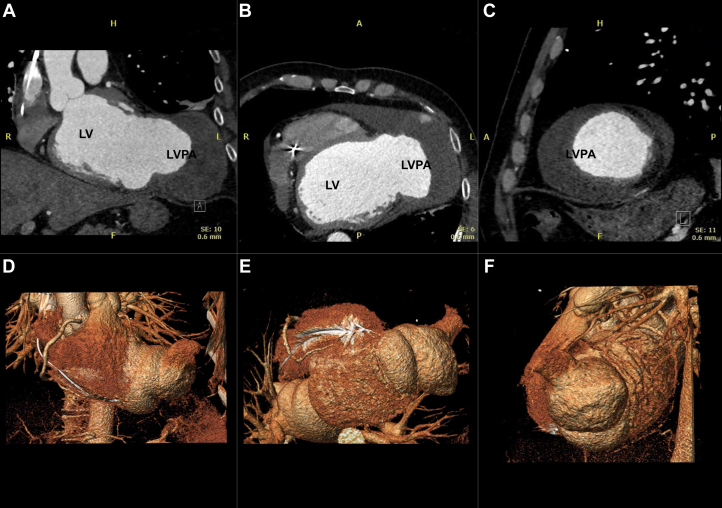
Dual-Source Cardiac Computed Tomography Angiography and 3-Dimensional Volume Rendering (A and D) Coronal views. (B and E) Axial views. (C and F) Sagittal views.

Dual-source contrast-enhanced cardiac computed tomography (CT) angiography (Figures 1A to 1F) revealed a 100 × 100 mm saccular dilation at the apex of the LV with a 70 × 60 mm neck, featuring a thin (2-3 mm) wall and late contrast enhancement. A large mural thrombus was identified, fissured to the cranial portion of the LV. Late contrast uptake also indicated a transmural scar in the aneurysmal segments and severe hypokinesis of the anterolateral wall.

**Figure 2:**
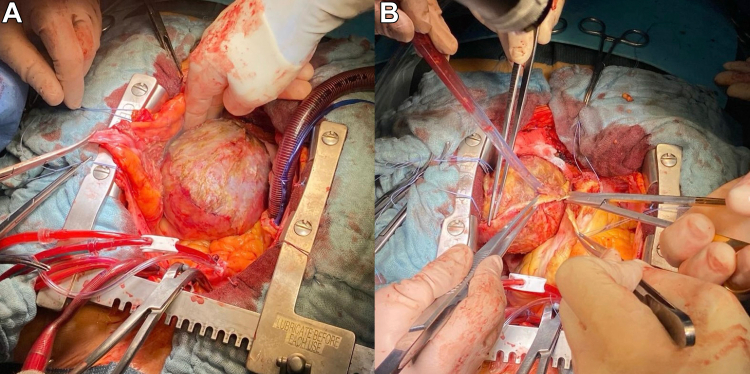
Intraoperative Views of Pseudoaneurysmal Mass (A) Pseudoaneurysmal mass closely adherent to the pericardium. (B) Surgical dissection revealing the mass during excision.

**Figure 3:**
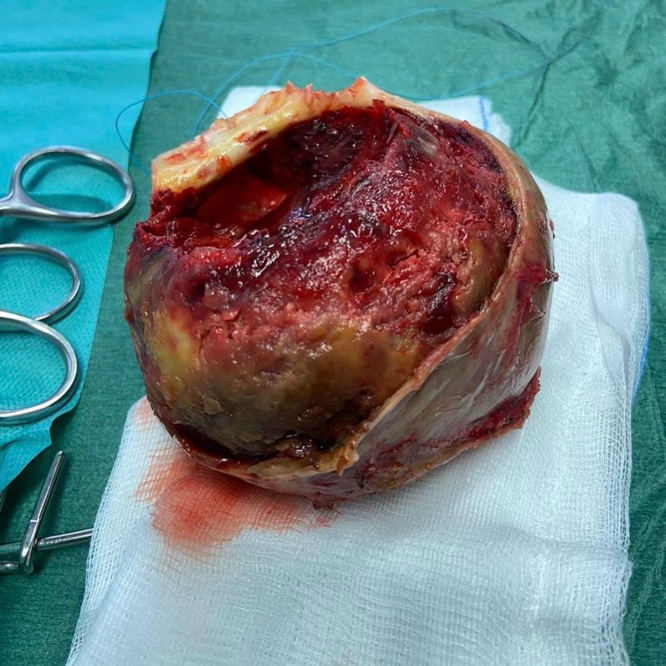
Large Removed Thrombotic Mass

**Figure 4:**
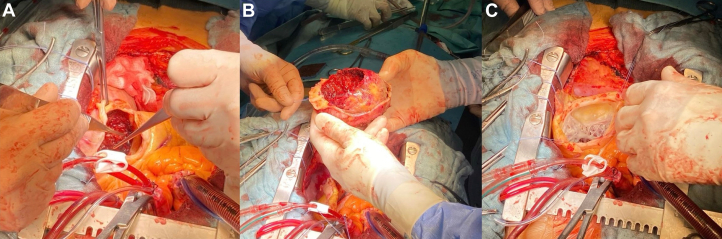
Intraoperative Findings and Thrombus Removal (A) Removal of the aneurysmal mass and associated clots. (B) Freshly removed giant thrombus. (C) Intraoperative view of left ventricular cavity through the apical incision, following evacuation of the pseudoaneurysmal thrombus.

Following discussion with the heart team, the patient was scheduled for LVPA removal via median sternotomy. On pericardial incision, the heart appeared markedly enlarged, with severely depressed contractility and a pseudoaneurysmal mass firmly adherent to the pericardium (Figures 2A and 2B). The apex was incised, and the pseudoaneurysmal cavity was evacuated, revealing a large thrombotic mass measuring 100 mm in diameter (Figure 3). Extreme care was taken to remove any residual clot (Figures 4A to 4C). Reconstruction of the left ventricular chamber was subsequently performed using a Dacron patch, following the Dor technique. The aorta was unclamped, and the heart resumed spontaneous activity in sinus rhythm. The patient was weaned off extracorporeal circulation with stable hemodynamic activity.

Predischarge TTE (Video 2) exhibited severe left ventricular dilation with moderate to severe systolic dysfunction, a left ventricular EF of 37%, and grade I diastolic dysfunction. At the 2-year and 3-month follow-up post-surgery, the patient demonstrated an EF of 35% and remained asymptomatic, with no signs of deterioration.

## Patient 2: Case Presentation

A 77-year-old woman presented to our department with NYHA functional class II symptoms and a posterolateral LVPA. Her medical history dated back 7 years to a posterolateral myocardial infarction caused by circumflex artery occlusion, complicated by ventricular free wall rupture. The initial rupture was surgically managed with infarctectomy and left ventricular wall reconstruction using a bovine pericardial patch reinforced with Teflon.

**Figure 5:**
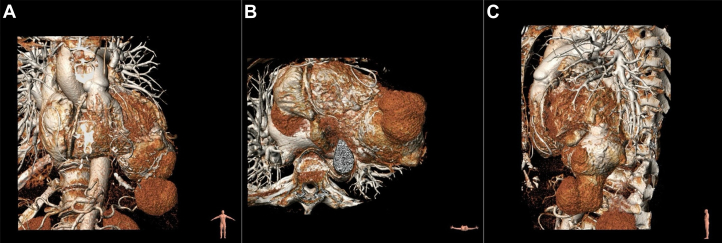
Computed Tomography 3-Dimensional Volume Rendering Reconstruction (A) Coronal view. (B) Axial view. (C) Sagittal view.

Five years after the initial surgery, routine echocardiography identified an LVPA at the site of the prior reconstruction, but the patient declined high-risk surgical intervention. One year later, she was admitted to the emergency department with left-sided subcostal pain. New transthoracic echocardiography performed almost 7 years after the AMI revealed severe left ventricular dilation with significant systolic dysfunction, including total hypokinesia of the inferior interventricular septum, basal inferior hypokinesia, and mid-distal inferior akinesia. Scarring and aneurysmal progression were noted in the lateral basal midsegment, along with a large, multilobular pseudoaneurysm (Video 3). Grade I diastolic dysfunction, mild-to-moderate functional mitral regurgitation, mild tricuspid valve regurgitation, and severe left atrial dilation were also observed. A subsequent CT scan confirmed the presence of a 95 × 75 mm LVPA protruding into the left hypochondrium, further illustrating the complications resulting from the previous myocardial infarction (Figures 5A to 5C).

The patient again refused surgery and underwent percutaneous exclusion of the LVPA via right transfemoral access under echocardiographic guidance, using a 40-mm Amplatzer Septal Occluder (Abbott).

Post-procedure TTE (Video 4) confirmed correct positioning of the device, with a small peridevice leak that did not result in any symptoms. However, owing to the posterolateral location of the lesion and limitations in the imaging window, the defect could not be fully assessed. Additionally, significant left ventricular dilation and severe global systolic dysfunction were observed.

At the 1-year 8-month follow-up, TTE showed a severely dilated left ventricle with an estimated EF of 40%, along with the previously treated large, partially thrombosed, multilobular pseudoaneurysm. Complete akinesia and scarring were observed in the posterior and lateral walls as well as in the basal inferior septum and mid-to-distal inferior wall. The Amplatzer device was partially visualized at the posterior and lateral wall level. Additionally, moderate mitral regurgitation was noted, showing slight progression from the previously reported mild-to-moderate degree.

**Figure 6:**
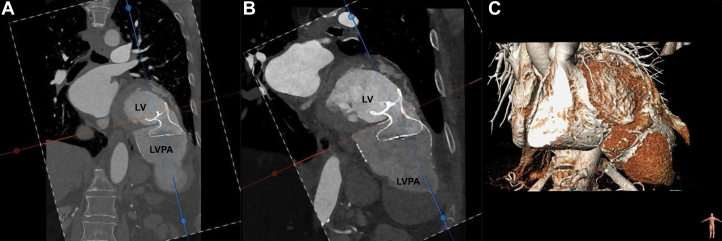
Coronal Cardiac CT (A) Postprocedural scan after percutaneous intervention. (B) Follow-up computed tomography (CT) scan. (C) Three-dimensional volume rendering reconstruction at follow-up. LV = left ventricle; LVPA = left ventricular pseudoaneurysm.

Subsequent CT revealed progressive enlargement of the pseudoaneurysmal sac by 30 mm compared with its previous size (Figure 6), leading to the onset of dyspnea and abdominal symptoms. Although the Amplatzer device initially provided adequate sealing, the persistent small leak—despite not increasing in size over time—likely contributed to enlargement of the sac owing to a possible flow passing through it, along with potential intradevice flow.

## Discussion

LVPA is a rare but serious complication following ventricular wall pseudorupture. Anterior wall rupture poses a greater risk of hemopericardium than posterior rupture. In contrast, posterior wall rupture usually induces inflammation, resulting in pericardial adhesions and pseudoaneurysm formation.^4^ Up to 50% of LVPA cases are diagnosed incidentally during follow-up imaging after AMI.^2^

The typical presentation of LVPA includes chest pain, peripheral embolism, life-threatening arrhythmias, and heart failure.^2^ Thrombus formation within a pseudoaneurysm requires specific conditions such as blood stasis, endothelial injury, and hypercoagulability. During the acute phase, thrombi may be in contact with the ventricular wall, but they can later become incorporated into the wall and undergo re-endothelialization.^3^ Approximately 30% of patients develop thrombi following an AMI, influenced by factors such as wall infarction, apical dyskinesia, and aneurysm presence.^5^ Echocardiography, cardiac CT, and magnetic resonance imaging provide detailed visualization of thrombus architecture, aneurysmal pouch, and surrounding structures in stable patients.^4^^,^^5^ Emergent or urgent surgery is considered the gold standard treatment for pseudoaneurysms owing to the high risk of embolization and rupture; however, surgical experience remains limited.^2^^,^^4^

These 2 cases are notable for the rare development of a large LVPA following myocardial infarction, along with the formation of substantial thrombotic masses. The size of the LVPA and thrombus are among the largest seen in our center clinical practice. In addition both patients had a rather uncommon delayed management of their LVPA, which was treated approximately 7 years after the AMI. Indeed, they survived for years with a large LVPA under medical management, whereas only a few cases of prolonged survival in this context are reported in the literature. Eventually both patients became symptomatic, and this progression is typically associated with a poor prognosis, including heart failure, suggesting the need for prompt intervention.^6^

Patient 1 presented with mild dyspnea as the only symptom, a surprisingly mild presentation given the severity of the underlying condition. Intraoperative findings revealed that the epicardial layer was barely containing the large thrombus, emphasizing the precarious nature of the case. Despite the high surgical risk, patient 1 successfully underwent surgery and continues to be monitored with regular follow-up.

In contrast, patient 2, who had previously declined surgical reconstruction, initially underwent percutaneous closure of the LVPA with apparent technical success. However, whereas percutaneous devices may appear optimally positioned at the time of placement, they can develop small leaks or other complications over time, potentially exacerbating the preexisting condition. The patient later returned with worsening symptoms, at which point surgical intervention was again offered but was declined owing to the high procedural risk. Further management will be guided by serial imaging and symptom progression.

Although the indication for surgery was clear in both cases, the optimal timing for surgery remains uncertain. Surgeons may consider a delayed repair approach in stable patients with acute LVPA, awaiting adequate healing of infarcted tissue, as this may reduce surgical risks. However, this approach carries significant drawbacks, including the potential for fatal rupture during the waiting period and the increased cost of extended intensive care unit stays. Conservative management may be appropriate for selected patients with small LVPA size and neck diameter.^6^^,^^7^

Percutaneous closure is an attractive option owing to its minimally invasive nature, but it does not allow for resection of the LVPA and the reconstruction of the left ventricle, owing to the anatomic and technical limitations of the transcatheter approach. These limitations can contribute to the worsening of the underlying condition over time. In contrast, surgical repair, although associated with higher risk, offers a more definitive long-term solution by directly addressing the aneurysm.

No studies currently compare the outcomes of surgical and percutaneous approaches, highlighting the need for further research. Although data on transcatheter LVPA closure appear promising, they may be influenced by several biases and by the variety of available devices. The true success rate remains uncertain, underscoring the need for prospective, multicenter studies to establish more definitive evidence.

## Funding Support and Author Disclosures

Dr Maisano has reported institutional grant and/or research support from Abbott, Medtronic, Edwards Lifesciences, Biotronik, Boston Scientific, NVT, Terumo, Venus, and Roche; has received consulting fees and honoraria, both personal and institutional, from Abbott, Boston Scientific, Medtronic, Edwards Lifesciences, Xeltis, Cardiovalve, Occlufit, Simulands, MTex, Venus, Squadra, Valgen, Croivalve, Meril, and Balmed; and has received royalty income and has held intellectual property rights with Edwards Lifesciences. He has been a shareholder (including share options) in Magenta, Transseptal Solutions, and 4Tech. All other authors have reported that they have no relationships relevant to the contents of this paper to disclose.

## Appendix

Video 1 Transthoracic Echocardiography With Biplane Imaging Four-chamber and 2-chamber views showing a large left ventricular apical pseudoaneurysm with a large mural thrombus.

Video 2 Transthoracic Echocardiography Postprocedural 4-chamber view showing the result of left ventricular pseudoaneurysm resection and left ventricular wall reconstruction with a Dacron patch according to the Dor technique.

Video 3 Transthoracic Echocardiography Three-chamber view showing a large, multilobar left ventricular pseudoaneurysm of the posterolateral wall in status postsurgical infarctectomy and left ventricular wall reconstruction.

Video 4 Transthoracic Echocardiography Postprocedural parasternal long axis view showing the result of percutaneous exclusion of the left ventricular pseudoaneurysm with the Amplatzer Septal Occluder.

## References

[1] Bohula E.A., Morrow D.A., Libby P., Bonow R.O., Mann D.L., Tomaselli G.F., Bhatt D.L., Solomon S.D. (2021). Braunwald’s Heart Disease: A Textbook of Cardiovascular Medicine.

[2] Lorusso R., Cubeddu R.J., Matteucci M., Ronco D., Moreno P.R. (2024). Ventricular pseudoaneurysm and free wall rupture after acute myocardial infarction: JACC focus seminar 4/5. J Am Coll Cardiol.

[3] Ben Jomaa S., Haj Salem N., Njima M., Zakhama A., Chadly A. (2020). Sudden death due to left ventricular thrombosis: a report of two autopsy cases. J Forensic Leg Med.

[4] Zouari F., Tlili R., Azaiez F. (2021). Thrombosed left ventricular pseudoaneurysm following myocardial infarction: a case report. J Med Case Rep.

[5] Fernández Cimadevilla O.C., Martín Fernández M., Santamarta Liébana E., Saiz Ayala A. (2010). A black mobile mass: diagnosis and management. Int J Cardiovasc Imaging.

[6] Torchio F., Garatti A., Ronco D., Matteucci M., Massimi G., Lorusso R. (2022). Left ventricular pseudoaneurysm: the niche of post-infarction mechanical complications. Ann Cardiothorac Surg.

[7] Duan Q.J., Duan C.T., Yang W.J., Dong A.Q. (2021). Conservative treatment of left ventricular pseudoaneurysm after mitral valve replacement due to early left ventricular rupture: a case report. J Cardiothorac Surg.

